# Swallowing sound evaluation using an electronic stethoscope and artificial intelligence analysis for patients with amyotrophic lateral sclerosis

**DOI:** 10.3389/fneur.2023.1212024

**Published:** 2023-08-03

**Authors:** Masahiro Nakamori, Ruoyi Ishikawa, Tomoaki Watanabe, Megumi Toko, Hiroyuki Naito, Tamayo Takahashi, Yoshitaka Simizu, Yu Yamazaki, Hirofumi Maruyama

**Affiliations:** ^1^Department of Clinical Neuroscience and Therapeutics, Hiroshima University Graduate School of Biomedical and Health Sciences, Hiroshima, Japan; ^2^Department of Dental Anesthesiology, Hiroshima University Graduate School of Biomedical and Health Sciences, Hiroshima, Japan

**Keywords:** electronic stethoscope, artificial intelligence, swallowing sound index, dysphagia, amyotrophic lateral sclerosis

## Abstract

**Background and purpose:**

Non-invasive, simple, and repetitive swallowing evaluation is required to prevent aspiration pneumonia in neurological care. We investigated the usefulness of swallowing sound evaluation in patients with amyotrophic lateral sclerosis (ALS) using our new electronic stethoscope artificial intelligence (AI) analysis tool.

**Methods:**

We studied patients with ALS who provided written informed consent. We used an electronic stethoscope, placed a Bluetooth-enabled electronic stethoscope on the upper end of the sternum, performed a 3-mL water swallow three times, and remotely identified the intermittent sound components of the water flow caused at that time by AI, with the maximum value as the swallowing sound index. We examined the correlation between the swallowing sound index and patient background, including swallowing-related parameters.

**Results:**

We evaluated 24 patients with ALS (age 64.0 ± 11.8 years, 13 women, median duration of illness 17.5 months). The median ALS Functional Rating Scale-Revised (ALSFRS-R) score was 41 (minimum 18, maximum 47). In all cases, the mean swallowing sound index was 0.209 ± 0.088. A multivariate analysis showed that a decrease in the swallowing sound index was significantly associated with a low ALSFRS-R score, an ALSFRS-R bulbar symptom score, % vital capacity, tongue pressure, a Mann Assessment of Swallowing Ability (MASA) score, and a MASA pharyngeal phase-related score.

**Conclusion:**

Swallowing sound evaluation using an electronic stethoscope AI analysis showed a correlation with existing indicators in swallowing evaluation in ALS and suggested its usefulness as a new method. This is expected to be a useful examination method in home and remote medical care.

## Introduction

Amyotrophic lateral sclerosis (ALS) is a neurodegenerative disease mainly affecting motor neurons. It causes progressive weakness, muscular atrophy, dysarthria, dysphagia, and dyspnea. Currently, there is no disease-modifying therapy available; therefore, it is important to manage nutrition, respiration, and communication. Swallowing dysfunction caused by bulbar dysfunction is a major factor in determining patient prognosis (1, 2). Aspiration pneumonia, caused by swallowing dysfunction, is a critical problem. Aspiration pneumonia is associated with high mortality rates in patients with ALS (3). The evaluation of swallowing function is important for assessing the risk of aspiration and monitoring the progression of neurological impairment. Videofluoroscopic or endoscopic examination is the gold-standard method for evaluating swallowing function. There is not much burden on the patient during the examination; however, limited facilities use these types of equipment. Therefore, a non-invasive screening method for evaluating swallowing dysfunction should be developed.

Simple and non-invasive instruments have been developed to evaluate the swallowing function. Tongue pressure measurement prevails (4–7). In addition, the usefulness of tongue ultrasonography for measuring tongue thickness and motion has been reported (7, 8). However, these methods primarily reflect only the oral phase of swallowing.

With the exception of videofluoroscopic or endoscopic examinations, there are few established methods for the evaluation of the pharyngeal phase (9). Recently, the evaluation of swallowing sounds, including deep learning analysis, has become the focus, and several types of instruments have been developed (10, 11). Especially, considering the anatomical aspects of swallowing sounds and their actual swallowing and reproducibility, commercialized and implementable tools have been introduced in the medical field. These methods have been used to predict the risk of aspiration and to assist in the selection of special diets provided to patients (11). We have combined electronic stethoscope techniques and artificial intelligence (AI) to assemble a system that incorporates sound recognition and swallowing sound quantification. In particular, the quantification of swallowing sounds using deep learning techniques provides objectivity and is a differentiating feature from traditional stethoscopes. Additionally, these systems do not necessarily require direct auscultation by medical staff as patients or caregivers themselves touch their own bodies, which makes remote medical checks possible. During a pandemic, these types of assessments are extremely useful and important.

This study aimed to investigate the usefulness of an electronic stethoscope for evaluating swallowing dysfunction in patients with ALS by comparing several disease parameters and Indicators of swallowing function.

## Materials and methods

### Standard protocol approvals, registrations, and patient consents

The study protocol was approved by the ethics committee of Hiroshima University Hospital (E-1599-1) and was in accordance with the guidelines of the 1964 Declaration of Helsinki. Written informed consent was obtained from all the patients or their relatives. All the data analyses were performed in a blinded manner.

### Participants

Consecutive patients with ALS who were diagnosed with definite, probable laboratory-supported ALS according to the revised El Escorial criteria (12) and admitted to Hiroshima University Hospital between 1 November 2021 and 31 December 2022 were enrolled in this prospective study. We excluded patients who could not tolerate full oral intake because of the risk of aspiration. Additionally, the swallowing sound index was evaluated using an electronic stethoscope and AI analysis of 57 healthy young volunteers.

### Swallowing test protocol

The swallowing sounds were recorded using an electronic stethoscope in a silent room. To exclude the effect of noise during the test, patients were given non-verbal instructions. The gestures were shown alone, and the patients were instructed to drink water. Each participant was seated on a chair with the back vertically fixed at 90°. Participants were instructed to drink water while sitting. The patients drank 3 mL of water. This was adopted based on the revised water swallowing test, one of the most commonly used screening methods for swallowing evaluation, which uses 3 mL of water. The water was at room temperature and measured with a 5-mL syringe (ss-20ESzp, Terumo, Tokyo, Japan), which was injected into the patient's mouth, after which a gesture was made for the patient to start swallowing.

Swallowing sounds were recorded in the 2 Hz to 20 kHz wavelength band using an electronic stethoscope (MSS-U10C; Pioneer, Tokyo, Japan) placed at the top of the sternum below the sternal notch. After exploring various sites for attaching the stethoscope such as the neck and chest, it was determined that the top of the sternum below the sternal notch, where motion artifacts are least likely to occur, is the most appropriate choice. The sound data were transferred to a waveform audio file format via Bluetooth, and the collected data were analyzed using a dedicated AI application.

### Swallowing sound evaluation using AI

In this study, a fine crackle sound discrimination AI algorithm for alveolar sound analysis was used to evaluate swallowing sounds, as previously reported (13). We have created algorithms not only for fine crackles but also for coarse crackles, wheeze, and rhonchi. Upon comparing and analyzing these sounds, we determined that the algorithm for fine crackles is the optimal choice. The AI analysis algorithm for calculating fine crackle sounds included 50 labeled sounds comprising characteristic frequency bands/continuations as teacher data for machine learning. Specifically, feature parameters (x) were extracted from the fine crackle sound caused by the inflow of water into the esophagus during swallowing using frequency, local variance, cepstrum analysis, the liftering process, and other methods. Next, the coefficients (a) of the feature parameters (x) were derived using AdaBoost as a machine-learning algorithm, and 50 pieces of labeled teacher data were added. The feature (y) was calculated using the following equation, consisting of the feature parameters (x) in the interval to be auscultated, and the coefficients (a) were determined using machine learning:


$$\begin{array}{l} y=\overset{148}{\underset{i=1}{\sum }}a_{i}x_{i}, \end{array}$$

*a*: Feature parameter coefficients calculated using machine learning

*x*: Feature parameter (normalized to −1≦ *x* ≦ 1).

The features (y) calculated for each frame at 12-ms intervals were converted to water inflow sound presence/absence data for each frame by comparison with a threshold determined using machine learning. The quantitative value of the fine crackle component (FCQV) was calculated for each second, based on the total number of frames and the number of frames in which the fine crackle sound was present.


$$\begin{array}{l} INDEX=\frac{No.\;of\;target\;sound\;frames}{Total\;frames\;in\;auscultation\;section}\times \;100 \end{array}$$

**Figure 1:**
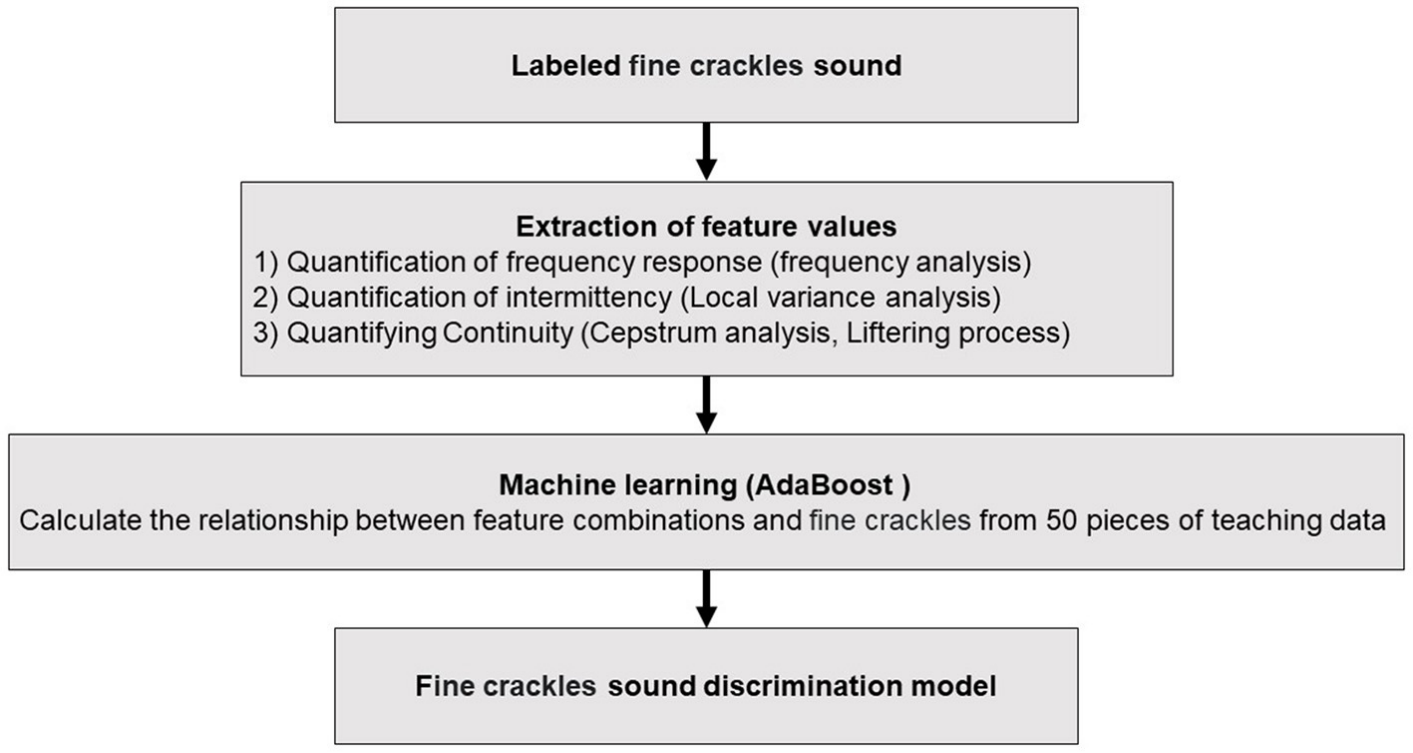
Algorithm for calculating swallowing sound index. The procedure for the swallowing sound analysis starts with the identification of fine crackle sounds, which are then subjected to feature value extraction through various techniques including frequency, local variance, and cepstrum analyses. These extracted features are fed into an AdaBoost machine-learning model, which is trained on 50 pieces of teaching data to discern the relationship between feature combinations and fine crackles.

In the present study, we used FCQV to perform acoustic analysis of water inflow sounds. When evaluating the analysis algorithm, a discriminator was created that discerned whether the target sound existed if the FCQV exceeded a certain value (Figure 1).

The electronic stethoscope had a contact-type pressure sensor in the diaphragm to switch it on and off at the start and end of the auscultation, respectively. Our system excluded 0.2 s immediately before and after the auscultation from the target auscultation section.

### Sample size

We calculated the required sample size based on a previous investigation of interstitial lung disease using an electronic stethoscope and AI analysis published by our research group (14). The minimum difference and standard deviation that we considered were 0.08 and 0.06, respectively. Based on an alpha level of 0.05 and a power of 0.80, we estimated that we would require a total of 20 participants.

### Data acquisition

The participants were functionally rated by neurologists using the ALS Functional Rating Scale-Revised (ALSFRS-R) (15). The ALSFRS-R is a validated questionnaire-based scale that measures physical function and performance of activities of daily living. The total possible score is 48, and lower scores correlate with increased disability. The ALSFRS-R is divided into five domains: bulbar-related (speech, salivation, and swallowing), upper limb-related (dressing and hygiene, turning in bed, and adjusting bed clothes), lower limb-related (walking and climbing stairs), and respiration-related (dyspnea, orthopnea, and respiratory insufficiency).

Tongue pressure was measured using a tongue pressure manometer equipped with a balloon probe (TPM-01; JMS Co. Ltd., Hiroshima, Japan). To measure the tongue pressure, the patients were asked to hold the cylinder so that the balloon could be placed between the tongue and the anterior part of the palate with the lips closed. Each subject was then asked to compress the balloon onto the palate for 7 s, three times at 1 min intervals. Measurements were performed as previously described (4, 16). The reliability of intraindividual measurements has been previously reported (17). The maximum value among the three measurements was considered as the tongue pressure for each patient.

We also performed a comprehensive swallowing evaluation using the Mann Assessment of Swallowing Ability (MASA) score. The MASA score is an established, concise, and comprehensive assessment tool that indicates the risk of swallowing dysfunction in patients with stroke (18). The MASA consists of 24 items with a total potential score of 200. It has also proven useful for various other diseases and types of patients with dysphagia (19). Furthermore, among the 24 items, pharyngeal phase-related eight items (gag, palate, cough reflex, voluntary cough, voice, tracheostomy, pharyngeal phase, and pharyngeal response), which account for 70 points, were evaluated.

These measurements were performed at the same time as electronic stethoscope recording.

### Statistical analysis

The data are expressed as mean ± standard deviation or median (minimum, maximum) for continuous variables and frequencies and percentages for discrete variables. Statistical analysis was performed using JMP 16 statistical software (SAS Institute Inc., Cary, NC, USA). The statistical significance of the intergroup differences was assessed using *t*-tests or χ^2^ tests, as appropriate. Baseline data of patients with ALS were analyzed, and two-step strategies were employed to assess the relative importance of the variables in their association with the swallowing sound index using multiple logistic analysis. First, a univariate analysis was performed. Subsequently, a multi-factorial analysis was performed with selected factors that had a *p*-value of < 0.05 in the univariate analysis and age. A *p*-value of < 0.05 was considered to be statistically significant.

## Results

**Figure 2:**
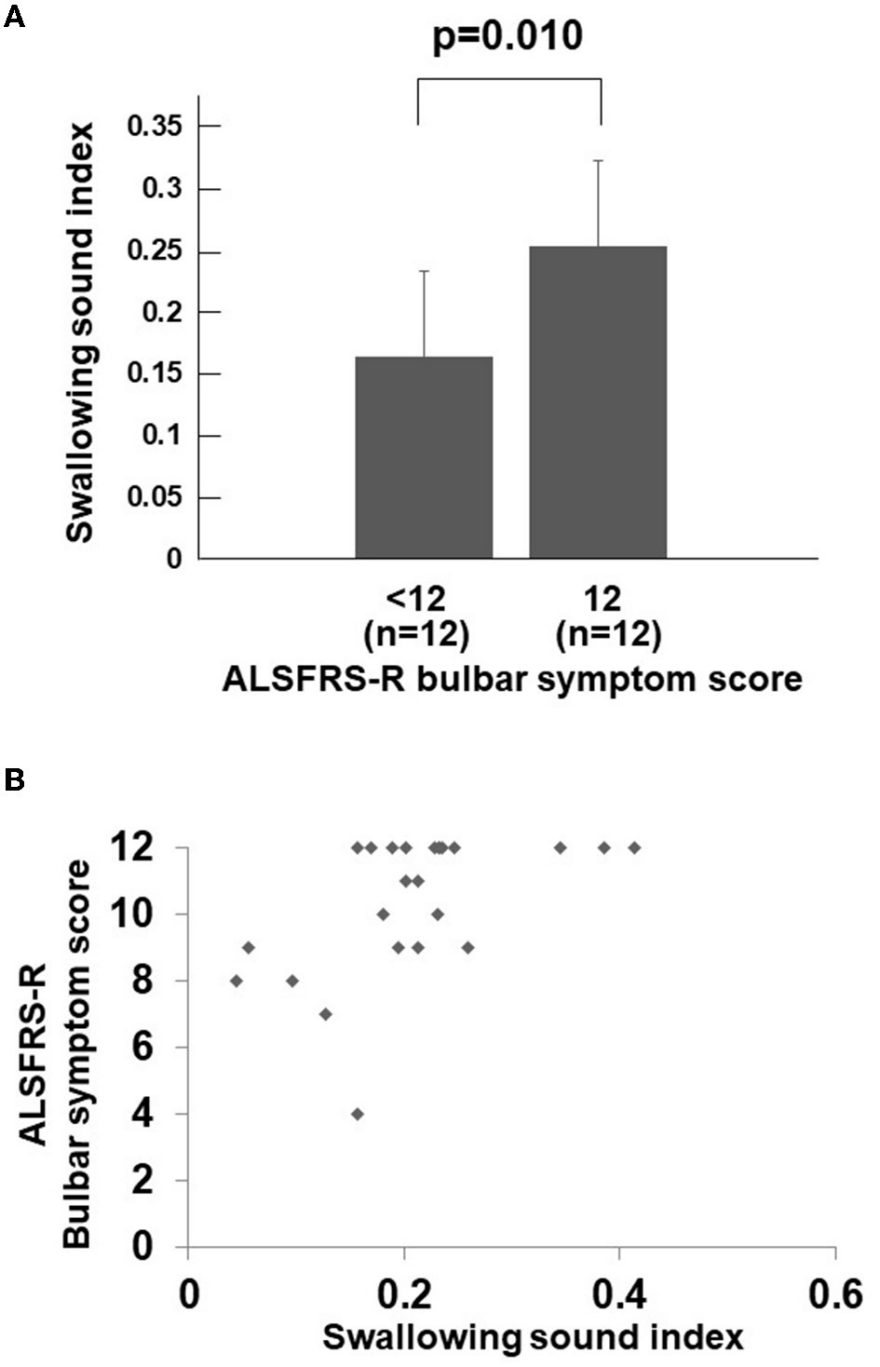
Swallowing sound index and ALSFRS-R bulbar symptom score. **(A)** Swallowing sound index between the ALSFRS-R bulbar symptom score <12 group and full score group. The swallowing sound index is significantly lower in the bulbar sub-score <12 group (*p* = 0.010). **(B)** Scatter plots of swallowing sound index and the ALSFRS-R bulbar symptom score. A positive correlation can be observed between the two, indicating a decrease in swallowing sound index with the bulbar symptoms. When the ALSFRS-R bulbar symptom score is less than 12 (full score), the swallowing sound index rapidly decreases. ALSFRS-R, Amyotrophic Lateral Sclerosis Functional Rating Scale-Revised.

During the study period, a total of 24 consecutive patients with ALS were investigated. Table 1 displays the background and physical characteristics of patients with ALS. The swallowing sound index measured and calculated using the same method as this study for 57 healthy young individuals (mean age 24.4 ± 1.9 years, 21 women, 36 men) was 0.369 ± 0.111, which was significantly higher than that of the patients with ALS (*p* < 0.001). The characteristics of the healthy young individuals are shown in Supplementary Table 1. Among the healthy young individuals, the mean swallowing sound index of men tended to be higher than that of women (*p* = 0.06); however, tongue pressure and body mass index were not correlated with the swallowing sound index (*p* = 0.84 and 0.58, respectively). The number of patients who complained of dysphagia was 11. Comparing the groups of the ALSFRS-R bulbar symptom score with a full score (*n* = 12) and < 12 (*n* = 12), the swallowing sound index of the group with a bulbar sub-score < 12 was significantly lower (Figure 2A). Scatter plots of the ALSFRS-R bulbar symptom score and the swallowing sound index are shown in Figure 2B. A positive correlation was observed between the ALSFRS-R bulbar symptom score and the swallowing sound index, indicating a decrease in swallowing sounds with the progression of ALS.

**Table 1:** Patients' background.

**Factors**	***N* = 24**
Age, year	64.0 ± 11.8
Sex (female), n (%)	13 (54.2)
Body mass index, kg/m^2^	21.9 ± 3.3
Duration from onset, month, median (minimum, maximum)	17.5 (4, 114)
ALSFRS-R score, median (minimum, maximum)	41 (18, 47)
ALSFRS-R bulbar symptom score, median (minimum, maximum)	11.5 (4,12)
Onset type	
Limb, n (%)	19 (79.2)
Bulbar palsy, n (%)	5 (20.8)
Serum albumin, g/dL	4.1 ± 0.4
% vital capacity	80.5 ± 20.4
Tongue pressure, kPa	27.6 ± 16.2
MASA score	195 (151, 200)
MASA pharyngeal phase-related score	70 (57, 70)
Swallowing sound index	0.209 ± 0.088

ALSFRS-R, Amyotrophic Lateral Sclerosis Functional Rating Scale-Revised; MASA, Mann Assessment of Swallowing Ability.

To explore the factors related to the swallowing sound index, a univariate analysis was performed on the factors listed in Table 1, revealing significant correlations with the ALSFRS-R score, the bulbar symptom score of ALSFRS-R, percentage of vital capacity (%VC), tongue pressure, and MASA (*p* < 0.05). Multivariate analyses were performed using individual factors and age. The results also revealed that the ALSFRS-R score, the bulbar symptom score of the ALSFRS-R, %VC, tongue pressure, the MASA score, and the MASA pharyngeal phase-related score were independently associated with the swallowing sound index (Table 2).

**Table 2:** Factors associated with the swallowing sound index.

	**Univariate analysis**	**Multivariate analysis**		
**Factors**	***p*** **-value**	**coefficient**	**95% CI**	***p*** **-value**
Age	0.065			
Sex	0.388			
Body mass index	0.492			
Disease duration	0.052			
ALSFRS-R score	0.009^*^	0.006	0.003 – 0.010	0.002^*^
ALSFRS-R bulbar symptom score	0.007^*^	0.019	0.003 – 0.035	0.022^*^
Onset type	0.169			
Serum albumin	0.18			
% Vital capacity	0.002^*^	0.002	0.001 – 0.004	0.003^*^
Tongue pressure	0.004^*^	0.003	0.001 – 0.005	0.009^*^
MASA score	< 0.001^*^	0.003	0.001 – 0.005	0.001^*^
MASA pharyngeal phase-related score	< 0.001^*^	0.011	0.005 – 0.017	< 0.001^*^

CI, confidence interval; ALSFRS-R, Amyotrophic Lateral Sclerosis Functional Rating Scale-Revised; MASA, Mann Assessment of Swallowing Ability. Multivariate analyses were performed using the individual factors, which showed a p-value of < 0.05, in univariate analyses and age. ^*^p-value of < 0.05.

Receiver operating characteristic (ROC) analysis was used to determine the cutoff value of the swallowing sound index that defined ALSFRS-R < 12, and a result of 0.228 was obtained (area under the curve =0.792, sensitivity 66.7%, specificity 83.3%, and *p* = 0.005).

## Discussion

In this study, we investigated the utility of a novel non-invasive and easily repeatable swallowing evaluation method, utilizing AI analysis of swallowing sounds via an electronic stethoscope, for patients with ALS.

Videofluoroscopic or endoscopic examinations are considered the gold standard for evaluating swallowing disorders; however, they carry high risks and can be challenging to perform in patients with advanced ALS who have respiratory dysfunction and other conditions. Therefore, non-invasive and easy-to-observe assessment methods have been explored. Tongue pressure and tongue ultrasonography are representative methods; however, they are limited in their ability to adequately evaluate the pharyngeal phase, such as pharyngeal residue, while reflecting the oral phase, such as oral passage and oral residue, of swallowing (7, 20).

Although the origin and clinical significance of swallowing sounds are not yet clearly understood, they gradually disappear as swallowing disorders progress due to the decrease in swallowing pressure. Decreased swallowing sounds are suggestive of swallowing disorders. It has been suggested that the origin of swallowing sounds is the sound of the bolus passing through the open esophageal entrance; as such, they are a measure reflecting the pharyngeal phase, which is not contradictory (21, 22). The swallowing sound index calculated only the bolus inflow sounds among the various noises produced during the pharyngeal phase and was independently associated with the MASA pharyngeal phase score in this study. The MASA pharyngeal phase score reflects swallowing reflex, pharyngeal elevation, and pharyngeal clearance (18). Thus, the swallowing sound index may be an indicator of pharyngeal phase dysfunction. In this study, AI analysis revealed that a decreased swallowing sound index in patients with ALS was associated with a low bulbar symptom sub-score. Previously, it was reported that a low bulbar symptom sub-score in ALS patients reflected the delay of oral transit time and pharyngeal transit time using videofluoroscopic examination (20). These results further supported the notion that the swallowing sound index reflects the presence of dysphagia in ALS patients. In addition, the swallowing sound index had a correlation with tongue pressure, an existing evaluation method. Tongue pressure has been also reported as a sensitive marker for the early detection of swallowing disorders in spinal and bulbar muscular atrophy, which is also a motor neuron disease (23). ALS, a representative motor neuron disease, is characterized by a widespread loss of motor neurons throughout the body (12). Therefore, it is anticipated that swallowing difficulties are widely affected in both the oral and pharyngeal stages (20). Given this, it is important to develop an instrument to detect a disorder of the pharyngeal phase, such as by measuring the swallowing sound index using an electronic stethoscope. Moreover, it was also shown that the swallowing sound index tended to decrease along with %VC. The respiratory function primarily relies on the diaphragm and intercostal muscles, and it is reasonable to interpret that the correlation between the swallowing sound index and respiratory function is due to the widespread loss of motor neurons throughout the body, leading to a parallel decrease in both measures. However, in basic research, many of the swallowing-related muscles are derived from the branchial arches, and studies suggest that respiratory neurons are activated during swallowing (24). Therefore, it is possible that the decrease in the swallowing sound index could lead to a decrease in %VC. Based on these results, the AI analysis of swallowing sounds using an electronic stethoscope can be a simple method for assessing swallowing function, including the pharyngeal phase.

The usefulness of electronic stethoscopes and AI analyses during the COVID-19 pandemic is highly regarded. Using a stethoscope, patients can record their own body sounds and remotely evaluate them, thus AI analysis is extremely meaningful in terms of infection prevention measures. Furthermore, home care and monitoring are crucial for patients with debilitating diseases, such as ALS. Therefore, the evaluation method introduced in this study is considered very useful and significant.

This study had several limitations. First, it was conducted at a single facility. ALS is a rare disease, making it difficult to conduct studies with a large number of cases; therefore, further studies involving multiple facilities and more cases are needed in future. Second, there has been no comparative study yet that includes the gold-standard videofluoroscopic examination. In patients with ALS, the videofluoroscopic or endoscopic examination itself carries the risk of aspiration; therefore, a careful selection of cases is required for its implementation. However, it is necessary to establish the usefulness of these new testing methods. It is important to optimize the interval of analysis using videofluoroscopic examination to improve the accuracy of swallowing sound evaluation.

Swallowing sound evaluation using an electronic stethoscope AI analysis showed a correlation with existing indicators in swallowing evaluation in patients with ALS and suggested its usefulness as a new method. It is expected to be useful for patients with neurological disorders and the elderly who are at risk of aspiration. Furthermore, it is expected to be a useful examination method for home and remote medical care.

## Data availability statement

The raw data supporting the conclusions of this article will be made available by the authors, without undue reservation.

## Ethics statement

The studies involving human participants were reviewed and approved by the Ethics Committee of Hiroshima University Hospital. The patients/participants provided their written informed consent to participate in this study.

## Author contributions

MN, RI, TW, MT, HN, and YS conceived and designed the study. MN, MT, TT, and YS performed the experiments. MN, TT, and YS analyzed the data. MN, RI, TW, MT, HN, and YS drafted the manuscript. YY and HM supervised the study. All authors contributed to the article and approved the submitted version.
