# Supplementary material for: Swallowing sound evaluation using an electronic stethoscope and artificial intelligence analysis for patients with amyotrophic lateral sclerosis
Source: Front Neurol. 2023 Aug 3;14:1212024. doi: 10.3389/fneur.2023.1212024 (PMC10435850; doi:10.3389/fneur.2023.1212024)
Supplement: Supplementary file 1 [file Table_1.docx]

**Supplemental Table 1 Normal controls’ background**

| Factors | n=57 |
| --- | --- |
| Age, year | 24.4±1.9 |
| Sex (Female), n (%) | 21 (36.8) |
| Body mass index, kg/m^2^ | 21.1±2.7 |
| Tongue pressure, kPa | 38.9±11.9 |
| Swallowing sound index | 0.369±0.111 |
